# Single Molecule Translation Imaging Visualizes the Dynamics of Local β-Actin Synthesis in Retinal Axons

**DOI:** 10.1038/s41598-017-00695-7

**Published:** 2017-04-06

**Authors:** Florian Ströhl, Julie Qiaojin Lin, Romain F. Laine, Hovy Ho-Wai Wong, Vasja Urbančič, Roberta Cagnetta, Christine E. Holt, Clemens F. Kaminski

**Affiliations:** 1 Department of Chemical Engineering and Biotechnology, University of Cambridge, Philippa Fawcett Drive, Cambridge, CB3 0AS UK; 2 Department of Physiology, Development and Neuroscience, University of Cambridge, Downing Street, Cambridge, CB2 3DY UK

## Abstract

Local mRNA translation occurs in growing axons enabling precise control of the proteome in response to signals. To measure quantitatively the spatiotemporal dynamics of protein synthesis in growth cones, we further developed a technique for single molecule translation imaging (SMTI). We report that Netrin-1 triggers a burst of β-actin synthesis at multiple non-repetitive sites, particularly in the periphery. The response is remarkably rapid starting within 20 seconds of cue application.

## Introduction

During neurodevelopment, the growth cone at the tip of a growing axon navigates to its remote target by sensing extracellular cues and responding to these guidance cues by turning, advancing, or pausing in a timely manner^1–3^. Given this complexity and the significant distance they travel, growth cones must possess a high degree of autonomy to react rapidly. Previous studies have provided evidence that local protein synthesis contributes to the fast and local responses in navigating axons^4–9^. Specifically β-actin, a key structural component of growth cones associated with their motility, was found to have its mRNA in axons^10^. β-actin translation in growth cones has been shown to occur within 5–10 minutes in response to an extrinsic cue, such as Netrin-1, and to polarise on the near-side of a gradient using immunocytochemistry and a live translation reporter^5, 9^. This represents a potential shift in the central dogma of receptor-based signaling and investigating the precise spatiotemporal dynamics of local translation at the single molecule (SM) level could provide valuable insights. For example, it is not known precisely where in the growth cone new proteins are synthesised, how fast synthesis occurs in response to a cue and whether it occurs repetitively in the same spot or singly in diverse sites indicative of polysomal versus monosomal translation respectively.

**Figure 1:**
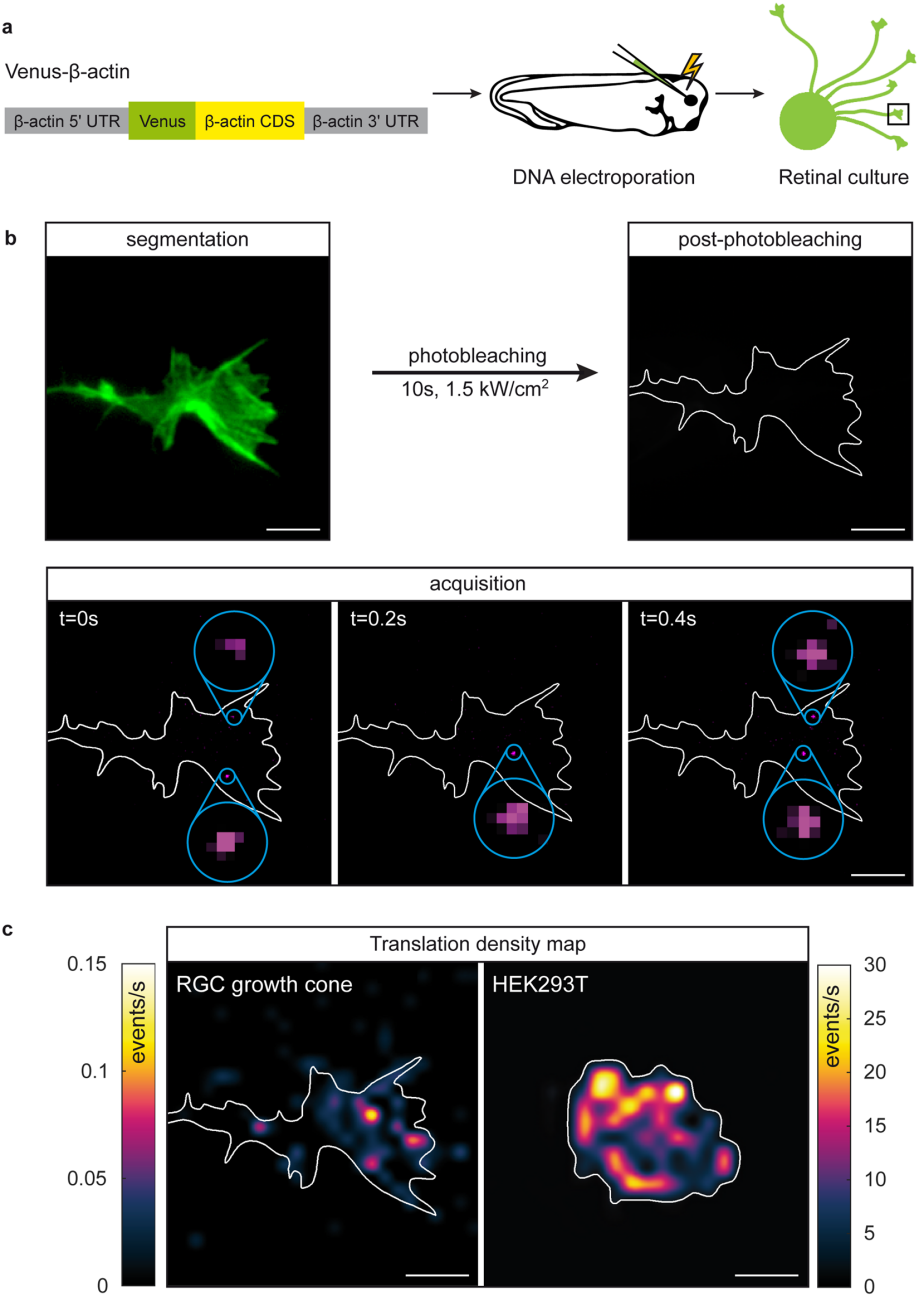
SMTI imaging procedure. (**a**) A reporter construct coding for Venus and a full-length β-actin sequence is electroporated into *Xenopus* eye primordia, which are dissected and cultured. (**b**) During imaging, a fluorescent image of a growth cone is acquired to segment the outline for later processing. Existing fluorescence is then photobleached using a brief pulse of laser irradiation. Subsequently, the translation of individual β-actin is recorded as individual Venus molecule emission. (**c**) Translation events are localized and processed to yield translation density maps of a representative RGC growth cone and a HEK293T cell. Scale bars are 5 μm.

Our original translation reporter approach^5^ does not allow for the translation events to be probed at the SM (protein) level. Recently, a number of techniques have been reported allowing spatiotemporal protein translation measurements with single-molecule resolution. Fluorescence amplification *via* multi-epitope tagging with *SunTag* proteins^11–13^ and site-specific dyes^14^ offer dynamic readout to visualize translation. Alternatively, a direct readout is also possible using single molecule translation imaging, SMTI^15, 16^. Here, we describe further developments of the SMTI approach through protocol optimization and analytical methods and demonstrate it to be a powerful tool for the quantification of translation dynamics in retinal ganglion cell (RGC) growth cones (Fig. 1).

## Results and Discussion

To measure *de novo* protein translation, the fast-folding and fast-bleaching fluorescent protein (FP) Venus^17^ is fused to the protein of interest, β-actin, flanked with the 3′ and 5′ untranslated regions (UTR) of β-actin. For our experiment, the DNA of the Venus-β-actin construct is introduced into *Xenopus* RGC neurons (Fig. 1a). As part of our SMTI protocol, existing fluorescence is bleached. Each newly synthesized Venus then emits a short burst of fluorescence within ~400 milliseconds before bleaching (Supplementary Fig. S1a). This fluorescence burst therefore signifies an individual translation event, which can be identified spatially via single-molecule localization procedures and temporally as part of a time-resolved acquisition (Fig. 1b). Hereby, rapid folding of the FP is essential^18^. The effect of photodamage inherent to this approach was investigated (Supplementary Fig. S1d) and all measurements were carried out within the window of low photodamage (<3 min). In baseline conditions, SM translation events were detected at an average rate of 10–20/min and located predominantly in the central domain. In contrast to HEK293T cells which exhibit ‘hotspots’ of repetitive Venus-β-actin SM translation during a 30 second period (Fig. 1c, left), or hippocampal dendrites where activity-regulated cytoskeletal-associated protein (ARC) and fragile X mental retardation protein (FMRP) undergo similar burst-like translation^15^, translation in RGC growth cones was sporadic with limited events reoccurring at the same location (Fig. 1c). The paucity of translational hotspots in axons could reflect growth cone motility and mRNA dynamics, or more intriguingly, the possibility of axonal translation predominantly by monosomes.

**Figure 2:**
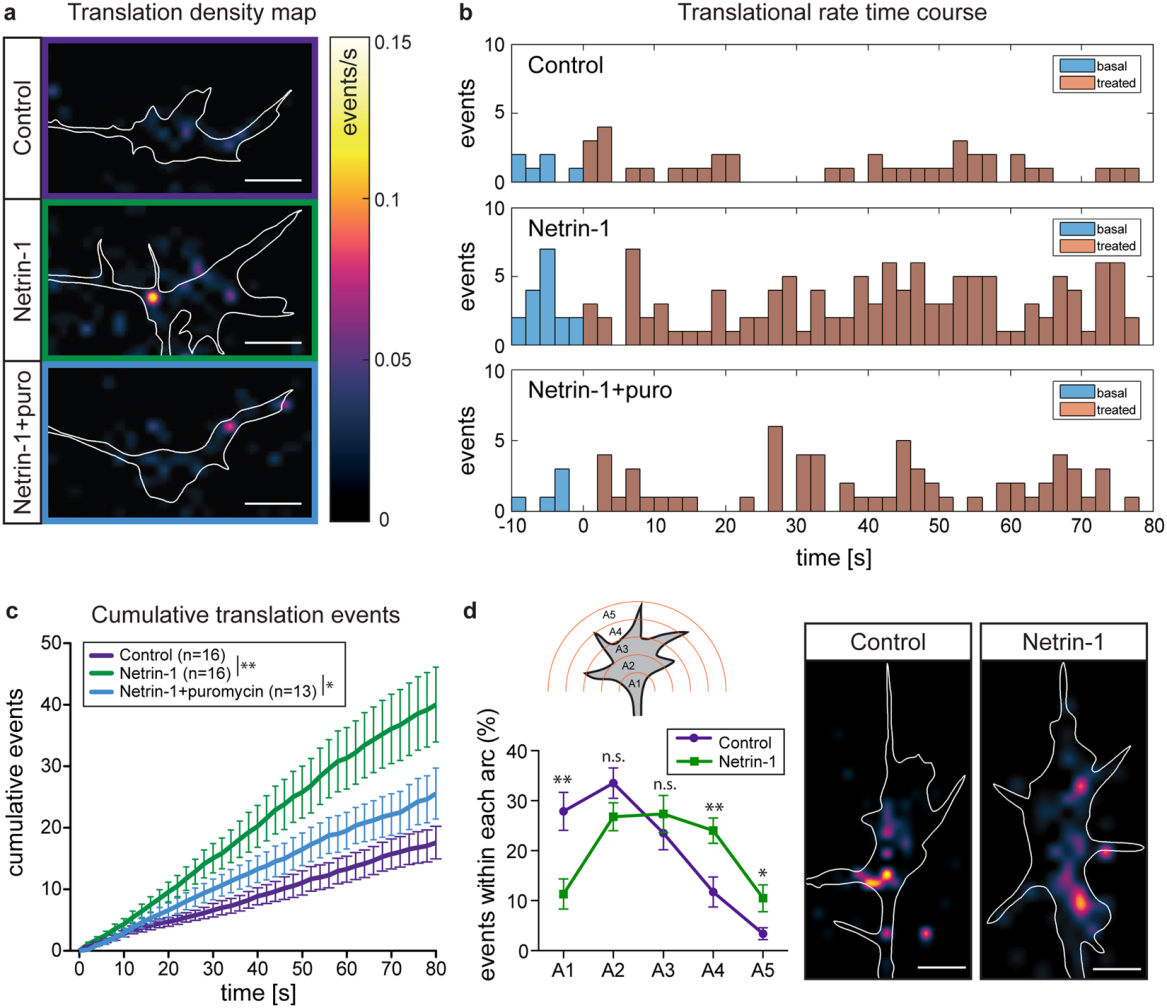
Netrin-1 increases β-actin translation rate. (**a**) Translation density maps for RGC growth cones treated with culture medium, Netrin-1, or Netrin-1 with puromycin pre-incubation. (**b**) Respective translation rate time courses of examples shown in **a**. The apparent difference in the pre-treatment rate between the culture medium- and Netrin-1-treated growth cones can be attributed to biological variability; the average pre-treatment rates between the two groups (n = 16) did not show any significant difference (p = 0.63). (**c**) Cumulative event rates per growth cone *p < 0.05; **p < 0.001; two-way ANOVA. (**d**) Sholl analysis on culture medium- or Netrin-1-treated growth cone density maps revealing the spatial distributions of translational events. The center of 5 concentric circles is located at the base of the growth cone, with the outermost circle intersecting the furthest boundary of the growth cone. The radii are equidistant. A1 denotes the most central arc, while A5 indicates the most peripheral arc. Intracellular events within each arc are counted and the percentages within each arc are shown in the graph. **p < 0.001; Mann-Whitney Houston test; scale bars are 5 μm. Error bars indicate standard error of the mean. In **c**, n is the number of growth cones.

Next we investigated the effect of Netrin-1 on β-actin translation in RGC growth cones. The analysis package we provide allows quantification and localization of the translation events across an acquisition as translation density maps, showing the event rate varying between 0–0.15 events/s (Fig. 2a). It also provides temporal information as a curve of instantaneous rate as a function of time. Remarkably, Netrin-1 stimulation led to a burst of β-actin translation starting ~20 seconds post treatment and lasting for ~30 seconds before gradually declining to lower, above baseline, levels (Fig. 2b and Supplementary Fig. S2a, Videos S5 and S6). The decline likely reflects the rapid desensitization of Netrin-1 receptors on the growth cone surface known to occur within 1–2 minutes^19^. The increase in translation is clearly visible in a cumulative translation events plot, with the baseline translation of around 15 events/min and the event number doubled within the first minute after Netrin-1 application (Fig. 2c). The observation of translation events was confirmed by pre-incubation of the samples with puromycin as a translation inhibitor, attenuating the Netrin-1-stimulated increase in translation (Fig. 2b and c, Supplementary Video S7). Incubation with puromycin also reduced the translation rate in HEK293T cells (Supplementary Fig. S2b,c,d) but had no significant effect in growth cones not stimulated with Netrin-1, which could be a result of the already low baseline translation overshadowing the effect of puromycin (Supplementary Fig. S3). To examine the spatial distribution of the translational events, we performed a Sholl analysis: Each growth cone was divided into five evenly spaced concentric arcs, labelled A1-A5 from central to peripheral. Arc A5 was set to circumscribe the outermost part of the growth cone. The percentage of β-actin translation events located in the central arcs decreased upon Netrin-1 stimulation, whereas translational events located at the growth cone periphery increased (Fig. 2d). This is in line with the observation of peripheral translocation of β-actin mRNA upon Netrin-1 stimulation (unpublished data). It has been reported that Netrin-1 stimulation leads to the disassociation of the Netrin-1 receptor DCC (Deleted in Colorectal Cancer) and promotes translation of the translational machinery. Consequently, this enhances protein synthesis in the vicinity of DCC, notably in the growth cone periphery^20^. The upregulation of growth cone peripheral translation detected by SMTI also matches the translocation of Venus-β-actin mRNA and is potentially a result of the increased availability of the DCC-associated translational machinery.

The SMTI methodology enables direct visualization of translation dynamics of individual, living cells with high spatiotemporal resolution. Using SMTI we were able to measure the effects of Netrin-1 on growth cones. We discovered that Netrin-1 transiently increases translation and found that it causes relocation of potentially monosomal translation sites to the growth cone periphery. This result is in line with a previously described shift in translation activity inside of growth cones, which were exposed to an external gradient of guidance cues^5^. However, SMTI also comes with limitations due to the comparatively high illumination intensities, which shorten the imaging duration of time-courses as phototoxic effects swiftly start to become problematic. Hence, for tracking of individual ribosomes over extended periods of time the *SunTag* methodology^11, 12^ is a more suitable alternative. However, as *SunTag* multi-epitope tagging requires labelling of a single protein with up to 50 fluorophores, a potential perturbation of cellular functions cannot be ruled out completely. In this respect SMTI is favourable as only a single FP is linked to the protein of interest. Also, the need for co-expression of multiple different proteins makes the *SunTag* translation imaging system far less straightforward to use than SMTI. Together with the SMTI analysis software package provided, SMTI is a ready-to-use technique for studying fast translation dynamics.

The most essential ingredient for successful SMTI is a both fast-folding and fast-bleaching reporter FP. In this study we built upon earlier work, which utilised Venus FP as it features a maturation half-time of under 2 minutes *in vitro* ^15^. Chaperones, enzymes and post-translational modifications possibly accelerate this process *in vivo*. Other fast-folding FPs like *folding reporter GFP* or the even faster *superfolder GFP* ^21^ are alternatives to Venus but are comparatively photostable. This often-desirable property of FPs is prohibitive for SMTI as effective photobleaching then requires higher, more phototoxic, illumination intensities. In terms of fast-bleaching, *mNeonGreen* ^22^ might be better albeit still being 10x more photostable than Venus. Here, certain red FPs like *eqFP611* ^23^ might offer a potential work-around. Upon translation *eqFP611* swiftly forms a green fluorescing intermediate before maturing into a red FP. Exploitation of this transient step in maturation might erase the need for fast bleaching. Unfortunately, new SMTI reporter proteins are developed at a slow pace due to the particular need for short folding times and low photostability, a requirement which is not in line with conventional aims of FP research. Nevertheless, more specialised probes will aid to the more widespread use of SMTI in future studies of high-speed protein translation dynamics.

## Conclusion

Using SMTI, we observed the rapid stimulating effect of Netrin-1 on β-actin local translation in RGC growth cones in real-time and the concomitant shift of its translation towards the periphery of the growth cones. Furthermore, we present protocol optimization (see methods) and analysis tools capable of exploiting the rich content of SMTI data. We note that the time at which the stimulation was observed (increase in event rate) is dependent on both the diffusion time of Netrin-1 in the solution and the variance in folding times of Venus. Better FPs featuring shorter folding and higher quantum yield may lead to improvements in the method’s temporal resolution and detection sensitivity. The ability to quantitatively measure local translation rates in space and time at the single molecule level in delicate cellular systems such as axonal growth cones paves the way to understanding signal-driven subcellular responses.

## Methods

### Embryos

*Xenopus laevis* embryos were obtained *via in vitro* fertilization and raised according to Leung *et al*.^24^ at 14–22 °C in 0.1x modified Barth’s saline (MBS; 8.8 mM NaCl, 0.1 mM KCl, 82 μM MgSO_4_, 0.24 mM NaHCO_3_, 0.1 mM HEPES, 33 μM Ca(NO_3_)_2_, 41 μM CaCl_2_). All animal experiments were carried out with approval of the *University of Cambridge Ethical Review Committee* according to the *University of Cambridge Animal Welfare Policy*.

### DNA constructs

Total mRNA extracted from stage 32 embryos using RNeasy Mini Kit (QIAGEN) was reverse-transcribed into a cDNA library with SuperScript III First-Strand Synthesis System (Thermo Scientific) using Oligo(dT) as primer. To obtain the Venus-β-actin fusion construct, full-length β-actin including the 5′UTR, the coding sequence and the 3′UTR obtained from the cDNA library by PCR was cloned into a pCS2+ vector with constitutive CMV promoter. The monomeric Venus coding sequence was inserted between β-actin 5′UTR and the coding sequence, followed by a short linker (KLEFK).

### Electroporation

Targeted eye electroporation was performed as previously described^25^. Eye primordial of stage 26 embryos were injected with DNA followed by electric pulses of 50 ms duration at 1000 ms intervals, delivered at 18 V. The electroporated embryos then recovered in 0.1X MBS.

### Primary retinal cultures

Glass bottom dishes (MatTek) were pre-treated with 5 M KOH (Sigma) for 1 hour, followed by 5 rinses with deionized water (Sigma). Eye primodia from stage 32–35 embryos were dissected and cultured in 60% L-15 at 20 °C for 18 hours (Invitrogen; Paisley, UK) on KOH-cleaned dishes coated with 10 μg/ml poly-L-lysine and 10 μg/ml laminin (Sigma; Gillingham, UK).

### HEK293T cell line and transfection

HEK293T cells were maintained in DMEM medium (ThermoFisher Scientific) supplemented with 10% FBS (ThermoFisher Scientific) in a 37 °C humidified incubator containing 5% CO_2_. Transfection was performed with jetPRIME (Polyplus transfection) following the manufacturer recommendations.

### Optical setup

Imaging was performed on a custom-made inverted single-molecule fluorescence microscope built around a commercial microscope frame (Olympus IX73). The illumination laser wavelength was at 488 nm (Coherent Sapphire) for excitation of the YFP derivate Venus in combination with a 525/45 emission filter (Semrock) and a dichroic beam splitter (Chroma ZT405/488/561/640rpc). The laser beam was circularly polarized to excite fluorescent proteins homogeneously regardless of their orientation. The microscope was equipped with an EM-CCD camera (Andor iXon Ultra 897) with effective pixel size on the sample of 118 nm. A 100x NA = 1.49 oil immersion TIRF objective (Olympus UAPON100XOTIRF) was used.

### Imaging sequence of HEK293T cell experiment

Prior to imaging, the HEK293T cells were bathed in 275 μM translation inhibitor puromycin (Sigma) for 30 minutes, or left untreated (control). The cells were then photobleached for 5 s with an irradiance of 1 kW/cm^2^ under episcopic illumination and Venus SMTI recorded with an exposure time of 10 ms for 25 s under TIRF illumination. We used the same intensity of 1 kW/cm^2^ for SMTI imaging as for bleaching due to the much higher translation rate in HEK293T cells near the nucleus. Imaging was performed with an additional EM gain of 200 to ensure single molecule sensitivity (Supplementary Fig. 2).

### Imaging sequence of primary retinal culture experiments

Prior to imaging, retinal cultures were pre-incubated for 30 minutes in conditions that mediate growth cone attraction to Netrin-1 (40 nM PP1 inhibitor tautomycin)^26^ and, in the negative control case, also pre-incubated with 275 μM translation inhibitor puromycin (Sigma). An outgrowing fluorescent growth cone was then selected and, prior to the bleaching step, imaged with low irradiance (<2 W/cm^2^) in both fluorescence and bright field mode to generate an outline image. The growth cone was then photobleached for 10 s with an irradiance of 1.5 kW/cm^2^. Afterwards, the flash-like recovery of Venus fluorescence was recorded with an exposure time of 200 ms for 60 s to generate a basal baseline. We used a reduced intensity of 0.3 kW/cm^2^ to ensure survival of the axons (Supplementary Fig. S1d) while simultaneously bleaching newly synthesized Venus. Then a drop of either 600 ng/ml Netrin-1 (Sigma) or culture medium as a control was added to the dish, while imaging was continued for another 120 s. After the total of 180 seconds of SMTI imaging another bright field image was taken to check for survival (an example is given in Supplementary Fig. S4). Retracted growth cones were excluded from analysis. All imaging steps were performed under epifluorescence illumination. An EM gain of 200 was used on the EMCCD camera to ensure single molecule sensitivity. The field of illumination was twice the size of the imaged field of view to bleach diffusing or transported FPs from outside the growth cone before entering the field of view.

### Data analysis

Localizations of individual protein translation events were retrieved using maximum likelihood estimation with a Gaussian model fit via the software package rapidSTORM^27^. A threshold of ~6700 ADC (Analog-to-Digital Counts), corresponding to ~500 photons per localization, was applied to filter out noise and non-Venus blinking events. This threshold was found by manual selection of Venus flashes and determination of the “average” photon budget of a single emitting Venus molecule (Supplementary Fig. S1c) and successive use as initial parameter settings for the rapidSTORM localisation software. Optimisation of the parameters was then performed on manually selected flashes as to recognise as many flashes as possible, while at the same time keeping the number of false positives as low as possible. This process was repeated by three independent researchers to minimize any bias occurring during the manual selection process. The tracking option of rapidSTORM was used to recombine photons emanating from the same FPs over multiple frames. In the growth cone experiments all events in a small area around the growth cones (rectangular windows that tightly crop the growth cones) were used for analysis due to the high mobility of filopodia. Results are given as translation events per second and with the standard error of the mean as error bar. In the HEK293T cell experiments, we distinguish between events happening inside and outside the cell by using an outline image generated from the SMTI acquisition and normalized the measured rate to the cell surface to ensure comparability of differently sized HEK293T cells. This was done via custom-written macros and software for ImageJ and MATLAB and yielded the translation rate density in translation events/s/μm^2^. Note that this area normalization step was not performed in the primary retinal culture assay as the objective’s depth of field was large enough to capture events from almost every axial position in the growth cones. The developed code for SMTI data analysis is freely available online under https://github.com/laseranalyticsgroup/SMTI.

### Data availability

Data are available upon request from the corresponding authors.

## Electronic supplementary material

Supplementary information

Supplementary Video S5

Supplementary Video S6

Supplementary Video S7
