# Supplementary material for: Single Molecule Translation Imaging Visualizes the Dynamics of Local β-Actin Synthesis in Retinal Axons
Source: Sci Rep. 2017 Apr 6;7:709. doi: 10.1038/s41598-017-00695-7 (PMC5428787; doi:10.1038/s41598-017-00695-7)
Supplement: Supplementary file 1 — Supplementary information [file 41598_2017_695_MOESM1_ESM.pdf]

SUPPLEMENTARY INFORMATION

Single Molecule Translation Imaging Visualizes the Dynamics of Local β-Actin Synthesis in Retinal Axons

Florian Ströhl[1,*], Julie Qiaojin Lin[2,*], Romain F. Laine[1], Hovy Ho-Wai Wong[2], Vasja Urbancic[2], Roberta Cagnetta[2], Christine E. Holt[2,$] & Clemens F. Kaminski[1,$]

[1]Department of Chemical Engineering and Biotechnology, University of Cambridge, Philippa Fawcett Drive, Cambridge, CB3 0AS, UK.

[2]Department of Physiology, Development and Neuroscience, University of Cambridge, Downing Street, Cambridge, CB2 3DY, United Kingdom.

[$]Correspondence should be addressed to C.F.K. (cfk23@cam.ac.uk) or C.E.H. (ceh33@cam.ac.uk)

[*]These authors contributed equally to the work

LEGENDS FOR SUPPLEMENTARY VIDEOS AND FIGURES

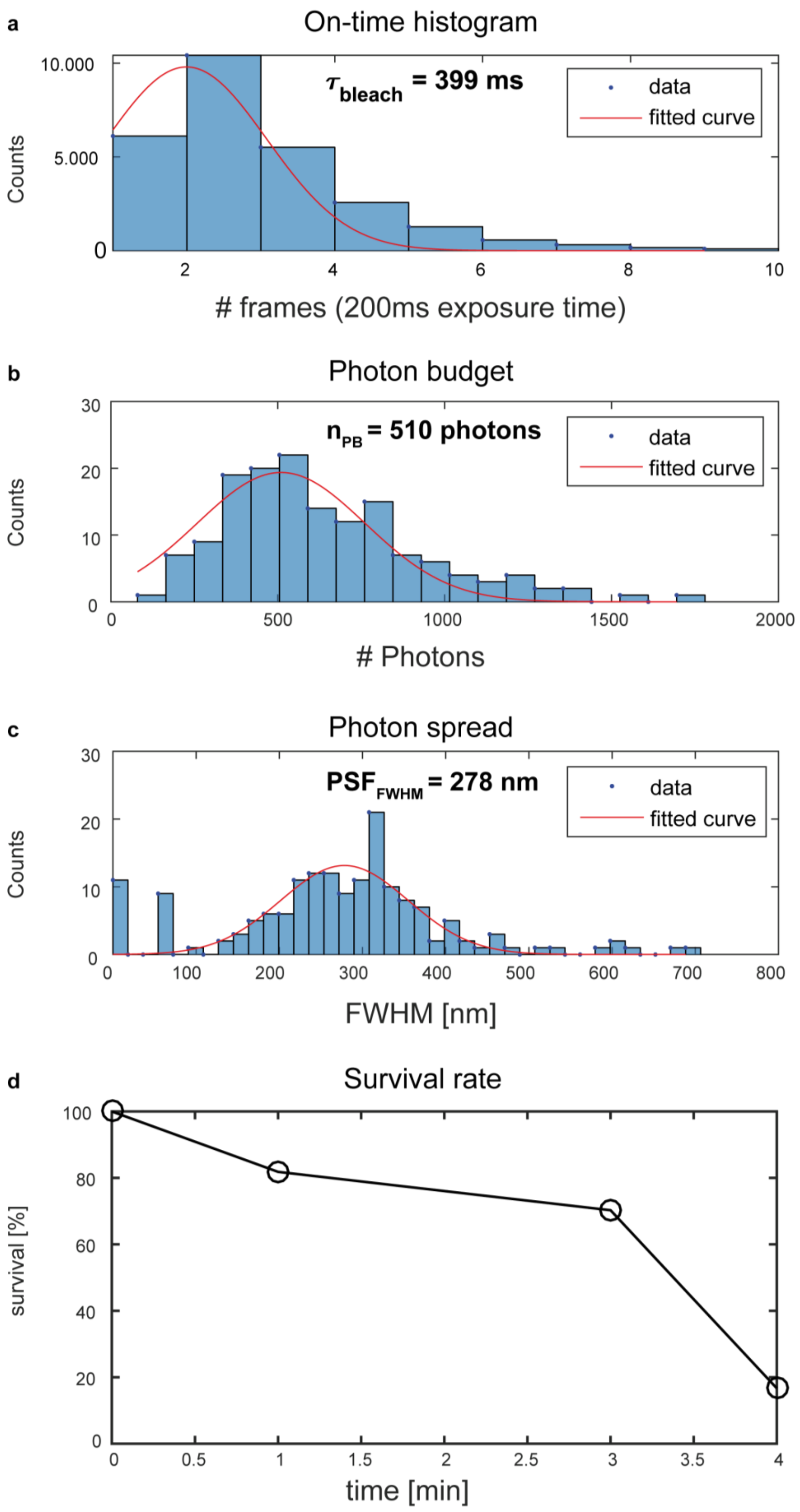

Supplementary Figure S1. Optimization and calibration of the SMTI setup. **(a)** To measure the light emitting “on-time” of the average Venus-β-actin molecule we SMTI-imaged RGCs containing the Venus-β-actin construct. This ensured optimization of the SMTI protocol within the experimental environment and conditions. We used a laser irradiation of 0.3 kW/cm^2 for continuous bleaching, which is the same as was used for determination of the growth cone survival time shown in (**d**). We imaged with 5 Hz frame rate and used rapi*d*STORM’s particle tracking capability (track emission) to determine the number of frames each flash was visible. Gaussian fitting of the “on-frame” distribution yielded an average “on-time” of τ_{bleach} = 399 ms before bleaching. To satisfy the Nyquist criterion we therefore chose a camera exposure time of 200 ms. **(b)** To differentiate between Venus flashes and non-specific blinks we determined the average photon budget of a Venus-β-actin molecule. The photon budget is the amount of photons a fluorophore emits before entering a non-fluorescing bleached state. We manually selected hundreds of flashes, which were subjectively classified as stemming from Venus emission. Using these as the ground truth we calculated that the average Venus-β-actin molecule emits 510 photons before bleaching. This corresponds to a localization precision of about 20 nm using the Thompson formula[1]. We chose this average value minus the half width at half maximum of the fitted distribution to be the minimum photon budget. Flashes detected by the rapi*d*STORM software below this threshold were not counted as Venus flashes and excluded from analysis. **(c)** For localization with rapi*d*STORM the expected size of fluorescent flashes should be known. We used the same data set as for the photon budget calculation and measured the average spread of photons by PSF fitting. The measured value is PSF_{FWHM} = 278 nm, which is slightly bigger than the theoretical size of a Venus PSF of 208 nm. This is due to out-of-focus flashes in the measurements, which widen the PSF. We therefore used the wider, measured, PSF as it supports localization of Venus flashes above and below the focal plane as is required for whole growth cone imaging. **(d)** After 3 minutes of continuous imaging with 0.3 kW/cm^2 about 30% of axons started to retract, while 4 minutes of imaging caused most axons to be retracted. 46 axons were observed for this plot.

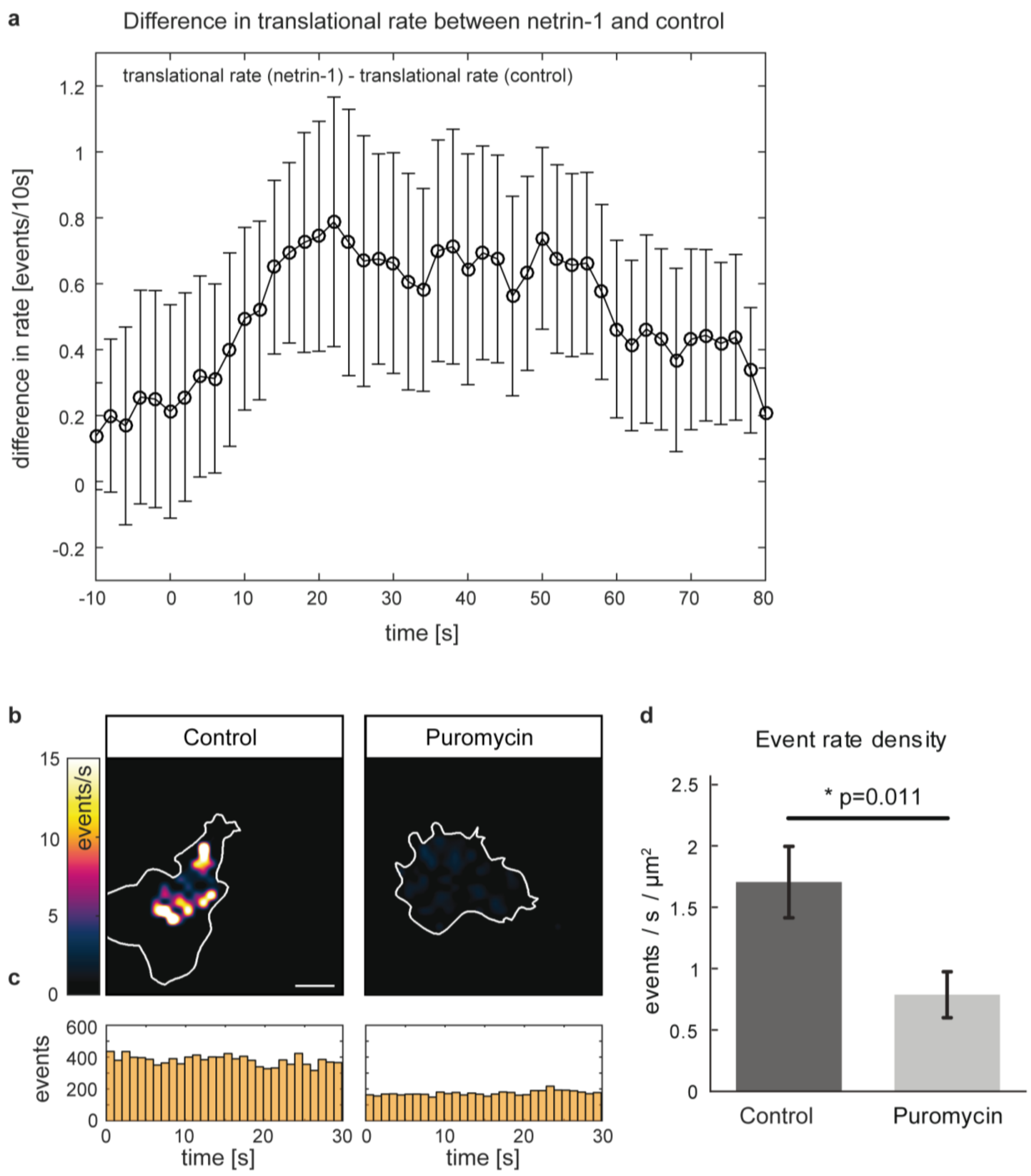

Supplementary Figure S2. Effect of Netrin-1 and puromycin on translation rates. (a) Difference rate of Netrin-1 and culture medium treated growth cones. Using Netrin-1 to stimulate growth cones leads to a transient peak in translation rate as compared to a control case with culture medium treatment. Treatment was performed at t=0. The peak is centered around 30 seconds post treatment and tails off around 60 seconds post treatment. Error bars

indicate standard error of mean. (**b-d**) Effect of puromycin on β-actin translation in HEK293T cells. (**b**) Examples of HEK293T cell translation density maps and (**c**) corresponding event rates. (**d**) HEK293T cell translation rate density in events/s/μm^2 of untreated and puromycin treated cells is significantly different. SMTI imaging was performed with TIRF illumination. Untreated (control) group n=20 cells, puromycin group n=20 cells. Scale bars are 5 μm.

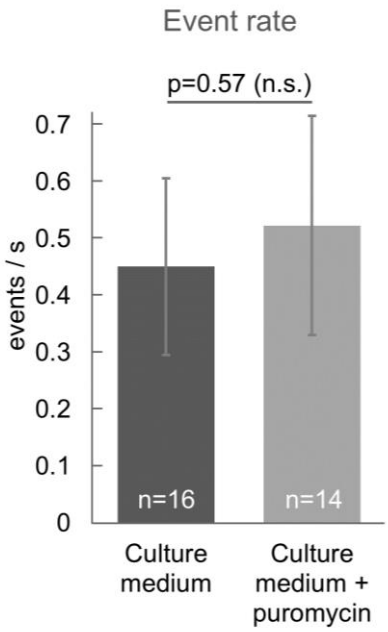

Supplementary Figure S3. Pre-incubation of growth-cones with puromycin did not show a significant effect in translations per second between non-incubated and puromycin-incubated growth cones (p=0.57). We used 16 non-incubated control growth cones and 14 puromycin-incubated control growth cones. Both groups also received a treatment with culture medium during SMTI imaging in accordance with the SMTI imaging protocol for growth cones. The already low number of translations in growth cones might overshadow the effect of puromycin. This result is also in line with previous publications[2,3], which showed that protein synthesis inhibitors did not completely inhibit protein synthesis upon cue stimulation or at baseline in growth cones.

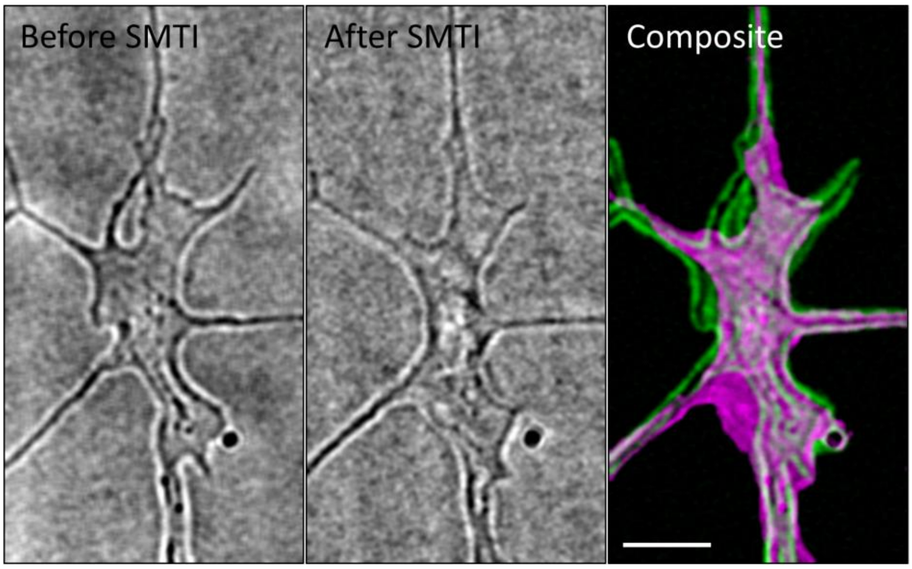

Supplementary Figure S4. Bright field images taken just before and right after SMTI imaging were used to check for growth cone survival. Furthermore, these images showed that the motion of growth cone bodies was limited, whereas filopodia were highly motile. Hence, a field of view around the growth cones was chosen for analysis, which takes movement of filopodia during SMTI imaging into account. Scale bar is 5 μm.

Supplementary videos

Three videos are provided to demonstrate the effect of various treatments upon protein translation and to show example SMTI data sets. The flashes visible outside of growth cones can be attributed to two main causes. (1) The flashes close to the growth cone outlines, which were indicted based on the initial fluorescence images, may be due to movement of growth cones and especially filopodia during imaging. These resulting small alternations of growth cone outlines were not captured in the imaging protocol. (2) The flashes further away from the growth cones are likely to be Venus molecules secreted by adjacent cells or released by dying cells in the dish. The density of extracellular flashes also increased upon poly-L-lysine and laminin coating of the culture dishes, indicating a possible contribution of the coatings chemicals to Venus molecule retention. The flashes observed outside of growth

cones are most likely Venus molecules, as cultured explants that did not express Venus protein did not show flashes intracellularly or extracellularly.

Supplementary Video S5. SMTI imaging of RGC growth cone treated with Netrin-1. Shown is the fluorescence outline image in the first tile, which was taken before SMTI recording, the raw data with the segmented outline overlaid in the second tile, the localized Venus flashes in the third tile (plotted cumulatively), and a histogram representation of detected Venus flashes under the other three tiles.

Supplementary Video S6. SMTI imaging of RGC growth cone treated with culture medium. Shown is the fluorescence outline image in the first tile, which was taken before SMTI recording, the raw data with the segmented outline overlaid in the second tile, the localized Venus flashes in the third tile (plotted cumulatively), and a histogram representation of detected Venus flashes under the other three tiles.

Supplementary Video S7. SMTI imaging of RGC growth cone treated with Netrin-1 after pre-incubation with puromycin. Shown is the fluorescence outline image in the first tile, which was taken before SMTI recording, the raw data with the segmented outline overlaid in the second tile, the localized Venus flashes in the third tile (plotted cumulatively), and a histogram representation of detected Venus flashes under the other three tiles.

REFERENCES

1. Thompson, R. E., Larson, D. R. & Webb, W. W. Precise nanometer localization analysis for individual fluorescent probes. *Biophys. J.* **82,** 2775–2783 (2002).
2. Leung, K.-M. *et al.* Asymmetrical beta-actin mRNA translation in growth cones mediates attractive turning to netrin-1. *Nat. Neurosci.* **9,** 1247–56 (2006).

3. Murakami, T. *et al.* ALS/FTD Mutation-Induced Phase Transition of FUS Liquid Droplets and Reversible Hydrogels into Irreversible Hydrogels Impairs RNP Granule Function. *Neuron* **88,** 678–690 (2015).
